# A System for Continuous Pre- to Post-reperfusion Intra-carotid Cold Infusion for Selective Brain Hypothermia in Rodent StrokeModels

**DOI:** 10.1007/s12975-020-00848-3

**Published:** 2020-09-10

**Authors:** Yi Wang, Jae H. Choi, Mohammed A. Almekhlafi, Ulf Ziemann, Sven Poli

**Affiliations:** 1 Department of Neurology & Stroke, Eberhard-Karls University of Tübingen, Tübingen, Germany; 2 Hertie-Institute for Clinical Brain Research, Eberhard-Karls University of Tübingen, Tübingen, Germany; 3 Graduate Training Center of Neuroscience, Eberhard-Karls University of Tübingen, Tübingen, Germany; 4 Neurological Surgery PC, Lake Success, NY USA; 5 Department of Clinical Neurosciences and Radiology, Calgary Stroke Program, Cumming School of Medicine, Hotchkiss Brain Institute, University of Calgary, Calgary, Alberta Canada

**Keywords:** Hypothermia, Selective brain cooling, Intra-arterial cold infusion, Ischemic stroke, Rodent model, Endovascular thrombectomy

## Abstract

Intra-carotid cold infusion (ICCI) appears as a promising method for hypothermia-mediated brain protection from ischemic stroke. Recent clinical pilot studies indicate easy implementation of ICCI into endovascular acute ischemic stroke treatment. Current rodent ICCI-in-stroke models limit ICCI to the post-reperfusion phase. To establish a method for continuous ICCI over the duration of intra-ischemia to post-reperfusion in rodent stroke models, a novel system was developed. Eighteen male Sprague-Dawley rats were included. Intraluminal filament method was used for transient middle cerebral artery occlusion (MCAO). Normal saline (~ 0 °C) was delivered (≤ 2.0 mL/min) into the internal carotid artery via a customized infusion system without interruption during MCAO (intra-ischemia) to after filament withdrawal (post-reperfusion). Bilateral cortical and striatal temperatures were monitored. Hypothermia goals were a temperature reduction in the ischemic hemisphere by 2 °C prior to reperfusion and thereafter maintenance of regional brain hypothermia at ~ 32 °C limiting the administered ICCI volume to ½ of each rat’s total blood volume. During ischemia, maximum brain cooling rate was achieved with ICCI at 0.5 mL/min. It took 2 min to reduce ischemic striatal temperature by 2.3 ± 0.3 °C. After reperfusion, brain cooling was continued at 2 mL/min ICCI first (over 42 s) and maintained at 32.1 ± 0.3 °C at 0.7 mL/min ICCI over a duration of 15 ± 0.8 min. ICCI (total 12.6 ± 0.6 mL) was uninterrupted over the duration of the studied phases. First system that allows continuous ICCI during the phases of intra-ischemia to post-reperfusion in small animals for selective brain cooling and for investigations of other neuroprotective infusions.

## Introduction

Despite strong pre-clinical results indicating the potential of therapeutic hypothermia to protect the brain in acute ischemic stroke [1, 2], clinical trials so far have failed to show a beneficial effect. Recent randomized controlled trials in stroke patients have struggled with feasibility and issues regarding the safety of whole-body hypothermia, the current standard of therapeutic hypothermia [3, 4].

**Fig. 1:**
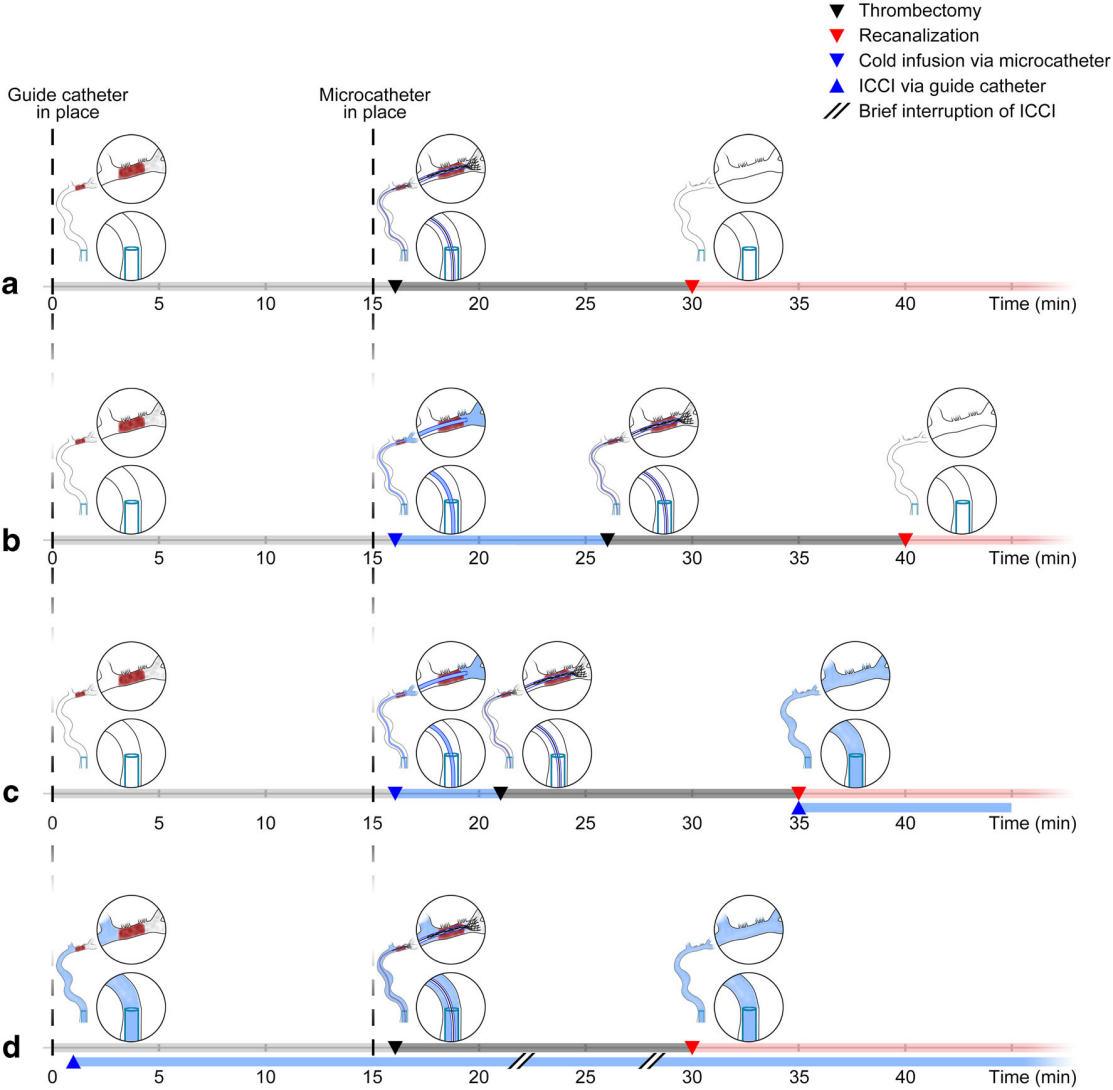
Cold infusion during endovascular thrombectomy (EVT). (a) The clinical workflow of EVT for treatment of acute ischemic stroke due to proximal vessel occlusion. (b) The implementation of intra-arterial cold infusion into EVT as performed in the clinical studies conducted by Peng et al. [9], where the microcatheter was used for pre-reperfusion cold infusion, and (c) the approach by Chen et al. [8] and Wu et al. [10] in which additional intra-carotid cold infusion (ICCI) was administered via the guide catheter after vessel recanalization. (d) The “guide catheter approach,” i.e., taking advantage of the guide catheter for ICCI as soon as it is placed in the internal carotid artery before recanalization. Simulation of the latter approach in a small animal stroke model was the aim of our study

As an alternative to whole-body hypothermia, selective brain cooling via cold infusion into brain-supplying arteries offers several advantages that could improve the likelihood of success to translate hypothermia-mediated neuroprotection from experimental studies into clinical ischemic stroke: First, by hijacking the cerebral circulation instead of fighting against the inflow of warm arterial blood into the brain, and targeting only the organ or tissue of interest, i.e., the ischemic hemisphere, this infusion method enables ultra-rapid induction of “focused” hypothermia [5]. Second, focused cooling allows adverse effects associated with whole-body cooling to be avoided, e.g., shivering and summit metabolism [6]. Third, with the increasing dissemination and utilization of endovascular thrombectomy (EVT; see Fig. 1(a)) [7], and by accessing the same EVT catheter pathways, implementation of intra-arterial cold infusion into the endovascular treatment regimen for acute ischemic stroke seems feasible. This is supported by three recent pilot studies where short-term local cerebral cold infusion was studied in acute ischemic stroke patients undergoing EVT [8–10].

In these clinical pilot studies, intra-arterial cold infusion was applied in two ways: In all three studies, the microcatheter was used to briefly infuse cold saline distally to the occlusion site, directly into the ischemic middle cerebral artery (MCA) circulation, and prior to actual thrombectomy. Cold infusion was then stopped for EVT (see Fig. 1(b, c)) [8–10]. Two studies [8, 10] additionally administered intra-carotid cold infusion (ICCI) through the guide catheter after completion of EVT (see Fig. 1(c)).

Unfortunately, none of the pilot studies could demonstrate any neuroprotective effect of cerebral cold infusion [8–10] and, despite promising animal studies [5], join the long queue of negative hypothermia-in-ischemic stroke trials. Only a change of the translational strategy might break this endless unlucky streak: prior to any further attempt of a human trial, treatment variables such as the optimal timing and duration of cold infusion, as well as the solution’s target temperature and composition, need individual attention, and technical aspects that may limit feasibility of future clinical implementation should be well addressed by the experimental study design and setup.

Theoretically, brain hypothermia that is established earlier to recanalization could more effectively mitigate the ischemic cascade and reperfusion injury and, ultimately, improve outcome [11, 12]. For instance, ICCI could be initiated much earlier in the process through the guide catheter that is placed into the internal carotid artery (ICA) already at the beginning of the EVT procedure (see Fig. 1) in order to bridge the interval until successful revascularization, which may take half an hour or even longer from guide cannula placement into the ICA [13, 14]. ICCI initiation would be even faster than the aforementioned microcatheter approach, albeit brain tissue cooling would be achieved not via direct infusion into the ischemic bed but via collateral pathways. Additionally, with the guide catheter infusion approach, the guide catheter remains free for deploying the micro-guidewire and the microcatheter, and the microcatheter itself remains available for EVT during ICCI which would avoid critical delay (“time is brain”; [15, 16]) of recanalization treatment as it has been accepted in all three clinical pilot studies (see Fig. 1(b, c)) [8–10]. The only difference to standard EVT is that the guide catheter is flushed by ICCI instead of non-chilled heparinized saline that is routinely used to prevent thrombus formation within the catheter. Finally, ICCI could be continuously applied from before to after recanalization with, if at all, only short interruptions during actual clot retrieval (in order to prevent distal embolization).

Our objective was to establish a novel infusion system for continuous pre- to post-reperfusion ICCI in small animal stroke models and simulate the clinical roadmap of EVT in the acute stroke setting (see Fig. 1(d)).

## Materials and Methods

### Study Design

This is a prospective, non-randomized, controlled, interventional experimental study.

### Setting and Animals

Animal facility was located and experiments were conducted at the Hertie Institute for Clinical Brain Research, Tübingen, Germany. Before conduct of experiments, male Sprague-Dawley rats (Charles River, Sulzfeld, Germany) were acclimatized in a specific-pathogen-free environment with 12/12-h reverse light-dark cycle and food/water provided ad libitum for at least 2 weeks.

### Anesthesia and Implantation of Probes

Anesthesia of spontaneously breathing rats was induced with isoflurane 5% (CP-Pharma, Burgdorf, Germany) (in room air), then maintained at 1.5–2% during operation. Premedication consisted of subcutaneously injected carprofen (5 mg/kg; Pfizer Deutschland, Berlin, Germany) and atropine (0.1 mg/kg; B. Braun, Melsungen, Germany); lidocaine (AstraZeneca, Wedel, Germany) subcutaneously was used for local anesthesia before any incision. Four thermocouple probes (diameter, 0.4 mm; AD instruments, Dunedin, New Zealand) were implanted in the striatum (bregma/lateral/depth: − 1/3/5 mm) and cortex (bregma/lateral/depth: − 1/3/3 mm) in both hemispheres for continuous monitoring of the brain temperature throughout the experiment. Laser Doppler flowmetry (PeriFlux 5000, Perimed, Järfälla, Sweden) was applied for regional cerebral blood flow monitoring (bregma/lateral: − 1/6 mm).

### Middle Cerebral Artery Occlusion

MCA occlusion (MCAO) was established with the filament method. Briefly, after dissecting the right common (CCA), external (ECA), and internal carotid arteries (ICA), ECA was ligated with 4–0 sutures (Ethicon, Norderstedt, Germany) as distally as possible. After clipping of CCA and ICA with micro-clamps (Fine Science Tools, Heidelberg, Germany), ECA was cut proximal to the ligation. A silicone-coated 4–0 filament (coating diameter/length 0.41–0.43/3–4 mm; Doccol, Sharon, MA, USA) was inserted retrogradely into the ECA, and the open end of the ECA was closed with another 4–0 suture. After removing the ICA clip, the filament was forwarded into the ICA and pushed forward 18–20 mm to block the opening of the MCA. Rats with cerebral blood flow drop to ≤ 40% of baseline value on laser Doppler flowmetry throughout the duration of MCAO were considered successful models (e.g., [17]). The filament was then secured with a clip around the ICA.

### Intra-carotid Cold Infusion for Regional Brain Hypothermia

**Fig. 2:**
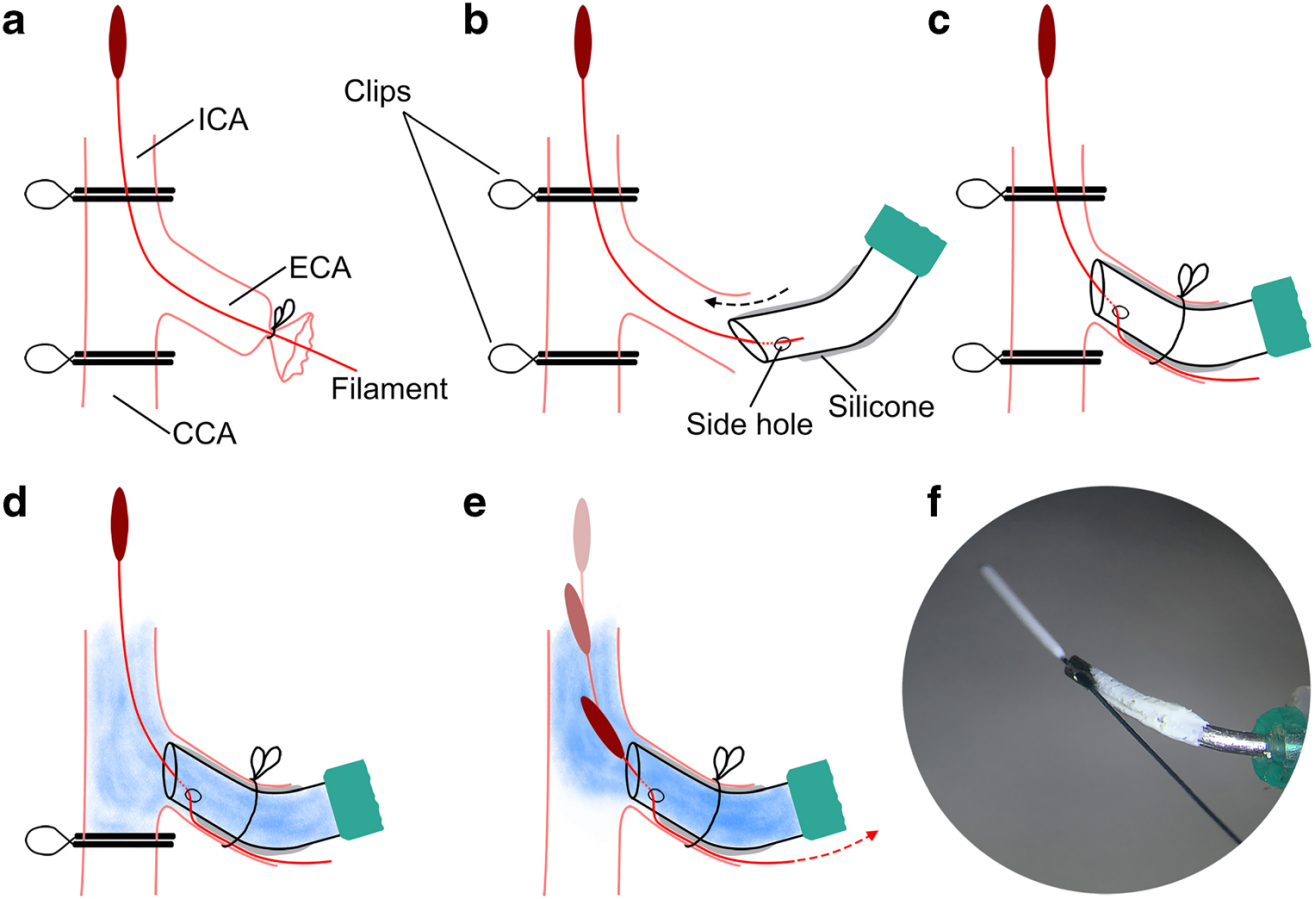
Infusion port. Illustration of the relevant experimental steps required for uninterrupted pre- to post-reperfusion cold intra-carotid cold infusion (ICCI). (a) The filament-induced middle cerebral artery occlusion (MCAO) model. (b, f) Our custom-made silicone-coated infusion port with a side hole that enables microsurgical insertion of the infusion port into the external carotid artery (ECA) during ischemia. (c) The infusion port in its final position. (d) Pre-reperfusion ICCI, followed—without interruption—by (e) intra- and post-reperfusion ICCI during and after retraction of the MCAO filament. CCA common carotid artery, ECA external carotid artery, ICA internal carotid artery

In order to allow for an uninterrupted ICCI (0.9% saline at an infusion temperature of 0–1 °C) from before to after reperfusion, we developed an infusion port (see Fig. 2) which permitted its placement during MCAO as well as filament retraction during ICCI. For this purpose, a 21G Safety-Multifly® cannula (Sarstedt, Nuembrecht, Germany) was bent, a side hole with a diameter of 0.3 mm was drilled in the cannula, and the tip was blunted and coated with elastic silicone (Soudal, Leverkusen, Germany) for vessel wall protection during insertion and prevention of leakage.

**Fig. 3:**
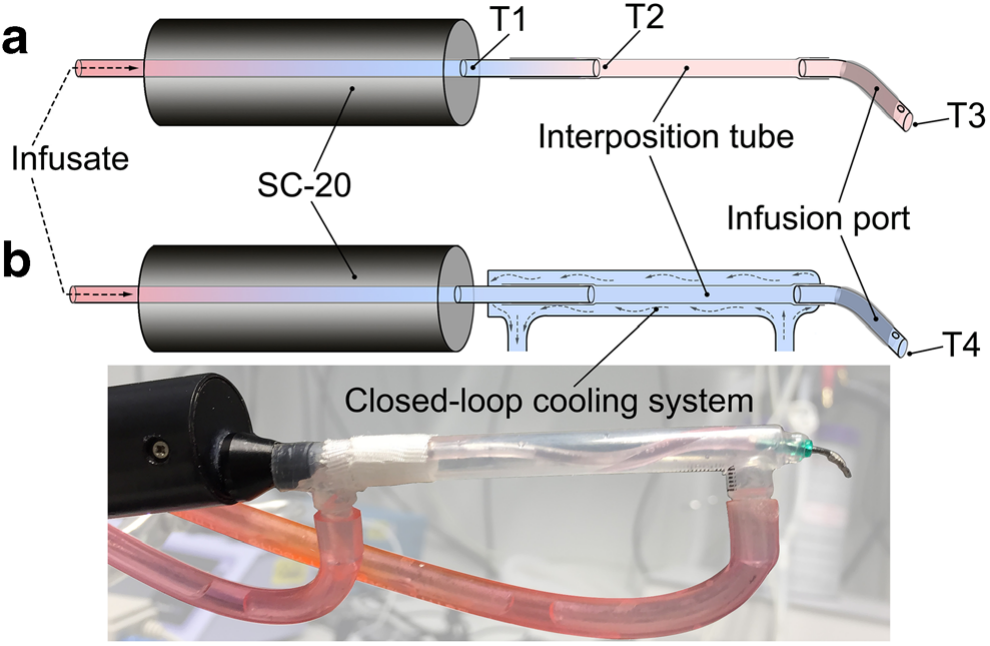
Infusate cooling systems. Black cylinders represent the commercially available SC-20 cooler from Warne Instruments (CT, USA) which was set to 0 °C (lowest temperature setting). In order to prevent re-warming of the infusate prior to entering the brain circulation of the rat, an additional custom-made closed-loop cooling system (shown in (b), sketch and photo) was incorporated to cool the line exposed to room air; the in-line cooling system was attached to a refrigerated bath circulator that was set to − 20 °C. T1 to T3 and T4 show infusate temperature measurements from different sites (a) without and (b) with the additional cooling system installed (see Table 1 for results). A polyvinylchloride interposition tube of 4 cm length (inner diameter: 1.3 mm; Sarstedt, Germany) allowed visual control during microsurgical insertion of the infusion port into the external carotid artery

Ten minutes before planned reperfusion, the ECA suture was opened. The end of the MCAO filament was placed through the side hole of the infusion port which was then introduced into the re-opened ECA, advanced to the carotid bifurcation, and fixed with a 4–0 suture around the ECA (see Fig. 2(b, c)). The infusion port was connected to a programmable automatic syringe pump (Pump 11 Elite; Harvard Apparatus, Holliston, MA, USA). A dual in-line SC-20 heater/cooler (Warne Instruments, CT, USA) and a custom-made closed-loop cooling system were interconnected to cool, first, the infusate and, second, the line exposed to room air in order to prevent re-warming of ICCI prior to entering the rats’ circulation (see Fig. 3). The SC-20 was set to 0 °C, its deepest possible temperature. The additional cooling system was attached to a refrigerated bath circulator Phoenix II/P2-C25P (Thermo Fisher scientific, Karlsruhe, Germany), which was set to − 20 °C.

Simultaneously with removing the ICA clip, which keeps the filament in its position, the automatic infusion pump was started for pre-reperfusion ICCI (see Fig. 2(d)). Reperfusion was established by retracting the filament and, immediately after, removing the CCA clip while ICCI continued without interruption (see Fig. 2(e)).

A rectal temperature-feedback-controlled T-1000 homeothermic system (CWE, CA, USA) was set to 37.5 °C in order to maintain rats’ physiological body core temperature.

### Intravenous Cold Infusion

The same infusion system was used for intravenous cold infusion into the right femoral vein, in order to exemplify brain cooling efficacy during whole-body cooling. However, the T-1000 homeothermic system was turned off during intravenous cold infusion.

### Experiments for Establishing an Optimal ICCI Protocol

The experiments comprised of three parts. Part I was to determine cooling efficacy of ICCI at varying infusion rates, 0.2, 0.5, 0.7, 1.0, 1.5, and 2.0 mL/min, during filament MCAO (intra-ischemic pre-reperfusion ICCI) and after filament retraction (post-reperfusion ICCI). Infusion rates were tested sequentially in each animal; each infusion rate was tested for 30 s, and brain temperature was required to return to baseline value before a new trial. In order to limit hemodilution, we decided for 2.0 mL/min as the maximal infusion rate, which equals ~ 50% of the physiological CCA blood flow in rats [18]. Part II was to test different ICCI regimens and determine a continuous pre- to post-reperfusion infusion protocol with the following targets: first, during pre-reperfusion phase, cooling the ischemic brain hemisphere by − 2 °C; second, achieving regional brain temperature of ~ 32 °C, the quickest possible; and third, maintaining the latter target temperature as long as possible with a maximum ICCI volume corresponding to half of the individual rat’s total blood volume, i.e., ~ 30 mL/kg body weight [19]. Part III was to assess feasibility and reproducibility of the final ICCI protocol when initiated at the very end of MCAO of 100-min duration and to exemplify brain cooling efficacy of intravenous cold infusion when using the same infusion protocol. During part III, femoral artery cannulation was established with polyethylene (PE)-50 tubing (inner diameter 0.58 mm, outer diameter 0.96 mm, Becton Dickinson, Franklin Lakes, NJ, USA) for blood sampling and heart rate and blood pressure monitoring during ischemia (at 50 min of MCAO) and immediately after cold infusion.

All animals (of all experimental parts of the study) were deeply anesthetized with isoflurane 5% at the respective experiment’s end (i.e., 5 to 10 min after end of treatment with cold infusion) and decapitated as soon as they stopped breathing, and brains were visually assessed for hemorrhagic complications.

### Data Acquisition, and Analysis

Data including laser Doppler flowmetry, rectal, and brain temperatures were recorded with LabChart 8 and PowerLab system (both AD instruments) and plotted with Prism 6 (GraphPad Software, San Diego, CA, USA). Blood was analyzed with an i-STAT system using CG8+ cartridges (both Abbott, North Chicago, IL, USA). Cooling efficacy was interpreted from the plotted graphs by determining the maximum temperature drop from baseline and calculating cooling rates. If not specified, all data are expressed as mean values ± standard deviation. Adobe Photoshop 21 (Adobe, San Jose, CA, USA) was used to create the artwork.

## Results

The 18 rats were aged 11 to 12 weeks and weighed 360–470 g. Four and nine rats were treated with ICCI in parts I and II, respectively. In part III, four rats received continuous pre- to post-reperfusion ICCI and one rat intravenous cold infusion using the same infusion protocol. One ICCI-treated rat died from subarachnoid hemorrhage (confirmed in autopsy) at the time of filament retraction. No other hemorrhagic complications were found.

### Setup of the Cooling System

The addition of the custom-made closed-loop cooling system (see Fig. 3(b)) prevented the infusate from re-warming before entering the rats’ circulation. The flow rate–dependent infusate outlet temperatures without (T3), and with (T4) this additional cooling system are reported in Table 1.

**Table 1:** Temperature of the infusate before entering the rat circulation

Infusion rate (mL/min)	T1 (°C)	T2 (°C)	T3 (°C)	T4 (°C)
0.2	0.1 ± 0.4	8.9 ± 0.7	18.5 ± 0.2	2.8 ± 0.4
0.5	– 0.4 ± 0.2	5.0 ± 0.4	11.0 ± 0.2	0.2 ± 0.2
0.7	− 0.5 ± 0.1	4.5 ± 0.4	9.3 ± 0.3	− 0.3 ± 0.3
1.0	− 0.6 ± 0.1	3.9 ± 0.1	7.4 ± 0.1	− 0.3 ± 0.3
1.5	− 0.6 ± 0.1	3.2 ± 0.3	5.6 ± 0.2	− 0.1 ± 0.3
2.0	− 0.3 ± 0.1	3.0 ± 0.3	4.6 ± 0.1	0.3 ± 0.3

Infusate temperatures within (T1) the SC-20 cooler (Warne Instruments), and at its outlet (T2), and at the tip of the infusion port without (T3) and with (T4) the additional custom-made closed-loop cooling system (cf. Fig. 3). Infusion rates range from 0.2 to 2 mL/min. Temperature data are reported as mean values ± standard deviation of 30 s of stable measurement

### Flow Rate–Dependent Brain Cooling Efficacy During Ischemia and After Reperfusion

**Fig. 4:**
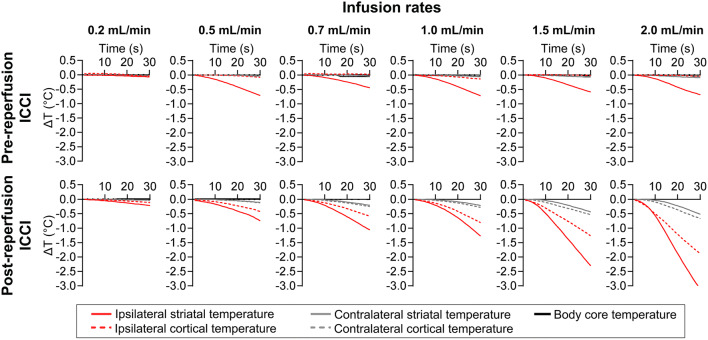
Flow rate–dependent brain cooling efficacy. Mean brain temperature decreases (∆*T*) during 30 s of pre- (upper row) and post-reperfusion (lower row) intra-carotid cold infusion (ICCI) at infusion rates from 0.2 to 2.0 mL/min. Infusion rates were tested sequentially in each animal; each infusion rate was tested for 30 s, and brain temperature was required to return to baseline value before a new trial

We observed different patterns of brain temperature decreases during 30 s of pre- and post-reperfusion ICCI (see Fig. 4). Whereas ICCI during MCAO only decreased ipsilateral striatal but not cortical temperature, post-reperfusion ICCI affected all measured brain areas, especially in the ipsilateral hemisphere. Furthermore, the cooling rate during pre-reperfusion ICCI plateaued at 0.5 mL/min but increased with higher infusion rates during post-reperfusion ICCI.

### Continuous Pre- to Post-reperfusion Infusion Protocol

**Fig. 5:**
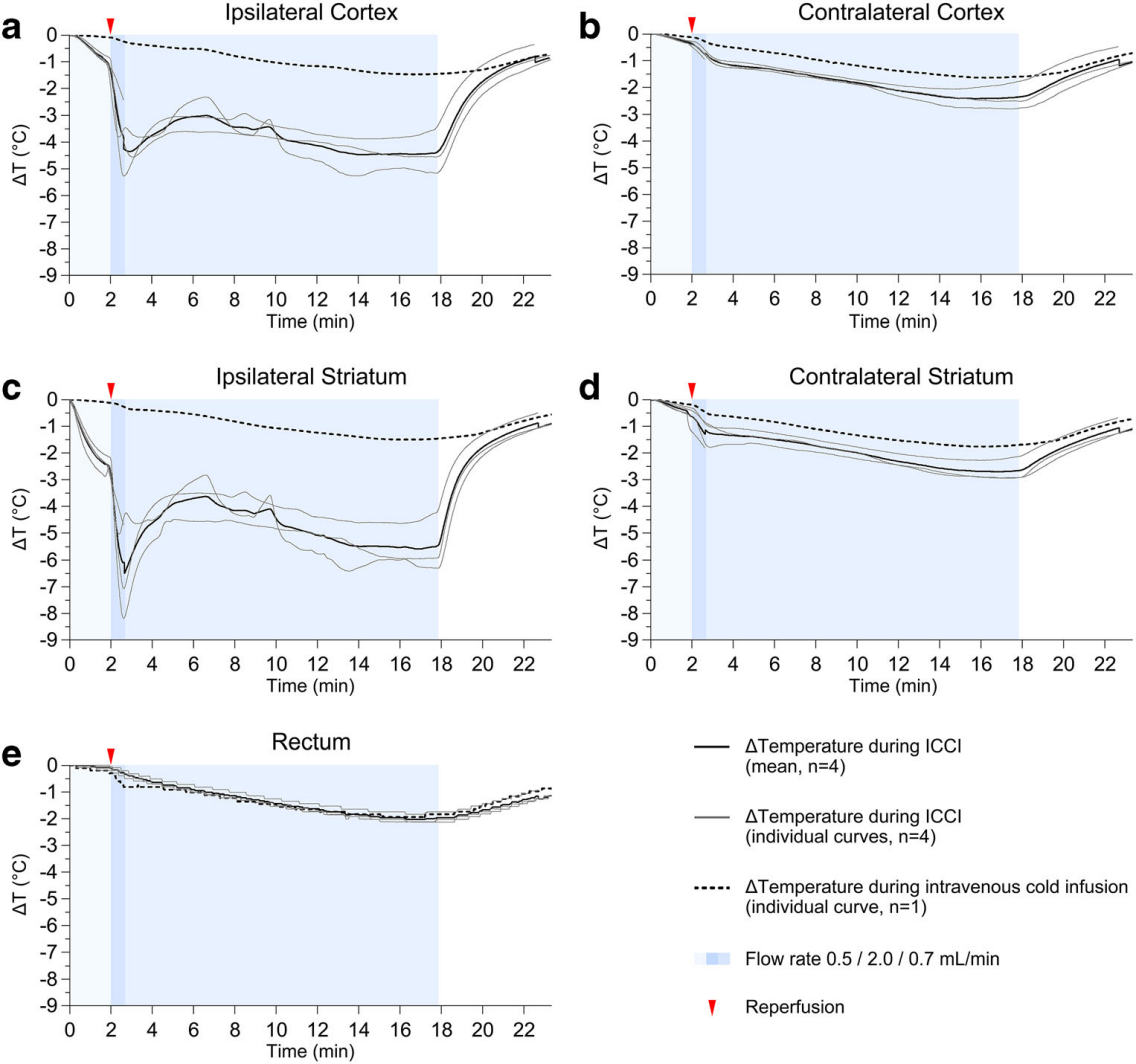
Brain temperatures and core body temperature during continuous pre- to post-reperfusion intra-carotid (ICCI) and intravenous cold infusion. Individual and mean temperature decrease (∆*T*) in the ischemic (a) and the contralateral (c) cortex, the ischemic (b) and the contralateral (d) striatum, as well as individual and mean decrease of core body temperature (e) during continuous pre- to post-reperfusion ICCI (*n* = 4) and intravenous cold infusion (*n* = 1). Goals: Final infusion rates and durations permit to reduce ischemic brain temperature by ~ 2 °C before filament retraction. To achieve and maintain the target temperature of ~ 32 °C in the ischemic tissue as quickly and as long as possible using a maximum infusion volume corresponding to half of the individual rat’s total blood volume

Considering the lack of infusion rate dependency of brain cooling during pre-reperfusion ICCI (see Fig. 4, upper row), we chose the lowest effective flow rate (i.e., 0.5 mL/min) in order to limit volume load. It took 2 min of 0.5 mL/min pre-reperfusion ICCI to reduce ischemic striatal temperature by 2.3 ± 0.3 °C before filament retraction (see Fig. 5).

Because cooling efficacy increased with higher infusion rates during post-reperfusion ICCI (see Fig. 4, lower row), we chose flushing with the maximal infusion rate (i.e., 2.0 mL/min) during filament retraction/reperfusion in order to reach the ~ 32 °C target temperature in the ischemic brain tissue as quickly as possible. Flushing with ICCI at 2.0 mL/min immediately after reperfusion over a duration of 42 s reduced ipsilateral brain temperature on average by 5.3 ± 1.8 °C compared with baseline (see Fig. 5).

Continued post-reperfusion ICCI at 0.7 mL/min permitted maintenance of regional hypothermia at 32.1 ± 0.3 °C in the ipsilateral hemisphere for a duration of 14.6 ± 0.84 min (see Fig. 5). On average, 12.6 ± 0.6 mL ICCI was infused over 17.3 ± 0.8 min.

Whole-body cooling with intravenous cold infusion revealed a much slower and less effective cooling of the ischemic brain tissue. Temperature changes obtained in the contralateral hemisphere and core body temperature were similar to those during ICCI (see Fig. 5).

Blood pressure, heart rate, and results of arterial blood gas analyses are displayed in Table 2. Initial and mean cerebral blood flow drop at start and during MCAO ranged from 12 to 27% and from 10 to 38% of baseline value on laser Doppler flowmetry.

**Table 2:** Results of arterial blood gas analyses and vital parameters during ischemia and after cold infusion in the four rats which were treated with intra-carotid cold infusion (ICCI) and the one treated with intravenous cold infusion

	ICCI		Intravenous cold infusion (*n* = 1)	
	After 50 min of MCAO (*n* = 4)	Immediately after ICCI (*n* = 3)*	After 50 min of MCAO	Immediately after intravenous cold infusion
pH	7.37 ± 0.02	7.32 ± 0.03	7.44	7.28
PaCO_2_ (mmHg)	50.0 ± 6.62	51.6 ± 4.16	37.4	55.7
PaO_2_ (mmHg)	97.3 ± 5.56	91.3 ± 4.04	108.0	91.0
Base excess (mmol/L)	3.5 ± 2.38	0.3 ± 0.58	1.0	0.0
Bicarbonate (mmol/L)	28.8 ± 2.57	26.4 ± 0.44	25.3	26.4
Total carbon dioxide (mmol/L)	30.3 ± 2.78	28.3 ± 0.58	26.0	28.0
Arterial oxygen saturation (%)	97.0 ± 0.82	96.0 ± 1.00	98.0	96.0
Sodium (mmol/L)	137.3 ± 1.50	140.7 ± 1.15	139	140
Potassium (mmol/L)	5.2 ± 0.61	4.7 ± 0.10	4.7	4.6
Ionized calcium (mmol/L)	1.4 ± 0.06	1.4 ± 0.09	1.34	1.45
Glucose (mmol/L)	12.2 ± 2.12	11.21 ± 2.71	11.8	11.7
Hematocrit (%)	39.8 ± 3.10	34.7 ± 3.21	42.0	36.0
Hemoglobin (mmol/L)	8.4 ± 0.66	7.32 ± 0.70	8.9	7.6
Mean arterial pressure (mmHg)	86.9 ± 12.96	78.1 ± 6.72	104	77
Hear rate (beats per min)	386.2 ± 55.33	319.6 ± 8.16	466	355

*One rat in ICCI group died at the moment of filament retraction

*pH* potential of hydrogen value, *PaCO*_*2*_ arterial carbon dioxide partial pressure, *PaO*_*2*_ arterial oxygen partial pressure

All data are reported as mean values ± standard deviation

## Discussion

We present the first experimental setup which permits continuous pre- to post-reperfusion ICCI for selective cooling of the ischemic hemisphere in a small animal stroke model. For a seamless translation from animal testing to bedside, our approach aimed at simulating the clinical EVT scenario in which the guide catheter is used for ICCI delivery as soon as it is introduced into the ICA, and ICCI is continued during EVT until after reperfusion of the ischemic brain tissue (see Fig. 1(d)).

### Comparison with Previous Experimental Setups

**Fig. 6:**
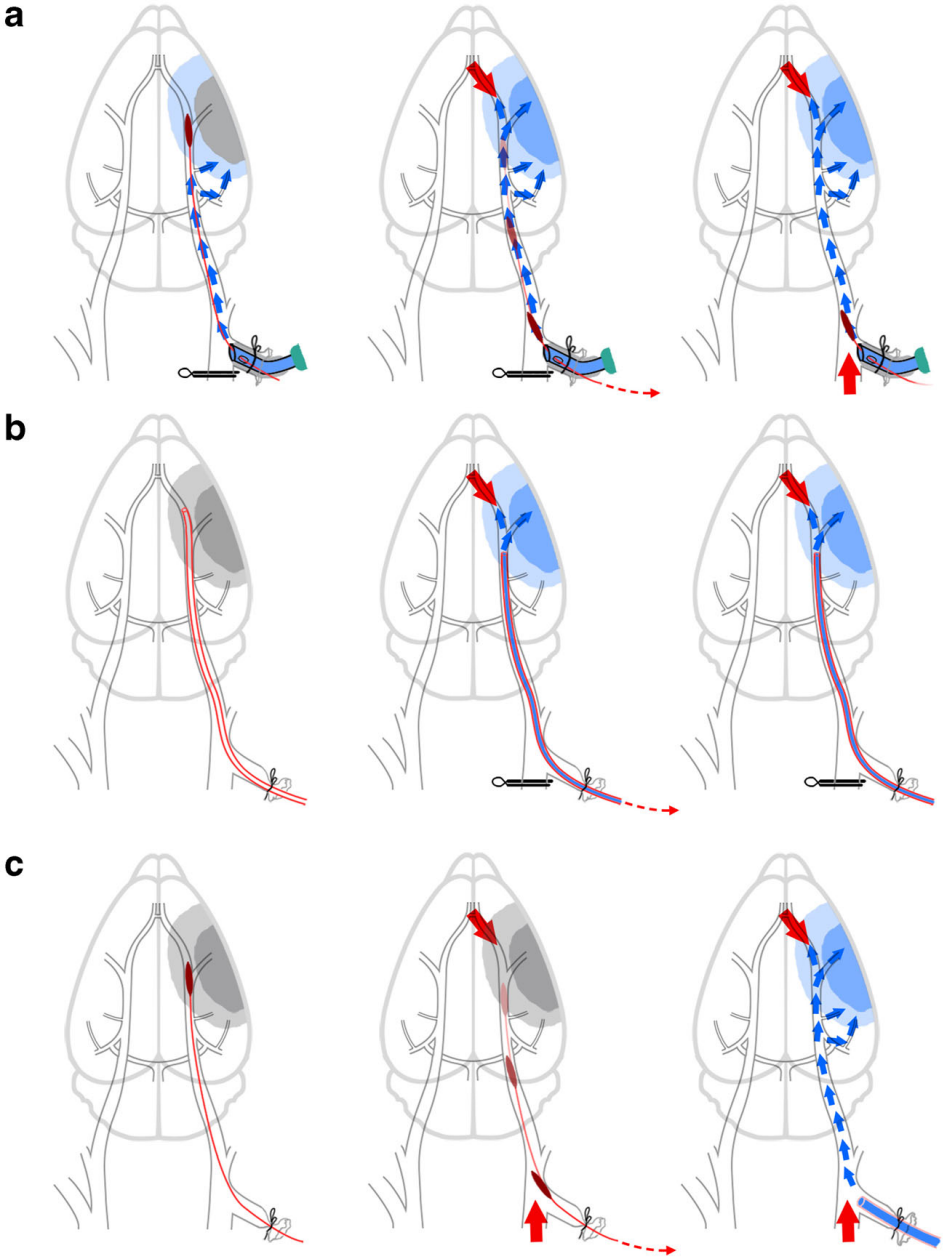
Existing experimental setups for testing intra-arterial cold infusion in small animal stroke models. (a) Our setup which currently is the only that allows for cooling of the ischemic brain tissue as early as during middle cerebral artery occlusion (MCAO) (left); intra-ischemic cold infusion reaches the ischemic tissue via collaterals (e.g., the anterior choroidal artery or leptomeningeal collaterals from the posterior cerebral artery); cold infusion is indicated by blue arrows. Furthermore, our setup allows for uninterrupted continuation of cold infusion during (middle) and after (right) filament retraction/reperfusion (see also Fig. 2); reperfusion is indicated by the red arrows. (b) In Ding’s approach (e.g., [20]), a modified PE-50 tube was used for both, MCAO (left) and delivery of cold infusion. Cold infusion was only started after collateral blood flow from the anterior cerebral artery was established, i.e., after tube retraction which was required in order to position its outlet upstream of the MCA opening (middle). (c) Ji’s approach (e.g., [21]) uses the same classic filament MCAO model as in the present study, but without the infusion port—thus, cold infusion in Ji’s experiment could only be initiated after completed reperfusion and with a delay of several minutes (right)

The current study shows that we were able to induce regional hypothermia already before MCA recanalization (see Fig. 6(a)). In contrast, previous small animal setups were limited to intra-arterial cold infusion in the post-reperfusion phase (see [5] for overview). Ding and colleagues used a modified PE-50 tube for both, MCAO and delivery of cold infusion (e.g., [20]). This tube is retracted in order to position its outlet upstream of the MCA opening which inevitably results collateral blood flow from the anterior cerebral artery entering the MCA territory before cold saline is administered (see Fig. 6(b)). Other researchers investigated ICCI in the conventional filament MCAO rodent model. In these, ICCI initiation was even further delayed after reperfusion because the tube intended for delivery of cold infusion could only be inserted into the ICA after withdrawal of the filament (see Fig. 6(c)) (e.g., [21]). This problem has been addressed in our study by integrating a dedicated infusion port.

In addition, (extracorporeal) re-warming of the cold infusate that is exposed to ambient air temperature is significant as observed in our experiment (see Table 1; Fig. 3(a)). The present results suggest that the brain cooling efficacy with ICCI is markedly higher in our experiment compared with that in previously reported studies because of the addition of an external in-line cooling system (see Fig. 3(b)) [5].

By allowing initiation of cold infusion before, during, and after reperfusion, and the possibility of controlling the infusate’s temperature, our approach is superior to the other experimental setups and especially predestined to be used for clarification of the optimal timing and duration as well as the optimal target temperature of cerebral cold infusion.

### Clinical Relevance of the Guide Catheter Approach

In the three clinical studies, the microcatheter was used for cold infusion during EVT [8–10]. Hereby, the microcatheter was navigated past the clot and cold infusion administered distally to the vascular occlusion site, thus, directly into the ischemic territory. This seems attractive for inducing pre-reperfusion hypothermia in the ischemic brain tissue. However, the use of the microcatheter for cold infusion leads to an inevitable delay of arterial recanalization, a critical delay that, if avoided, could save brain tissue from infarction and improve the chances for favorable outcome. In the previous clinical studies, this delay ranged from 5 (see Fig. 1(c)) [8, 10] to 10 min (see Fig. 1(b)) [9]. Furthermore, cold infusion needs to be stopped while thrombectomy devices occupy the microcatheter (see Fig. 1(b, c)) leading to fast potentially harmful tissue re-warming [22]. Besides, heat insulation of the microcatheter will remain an issue as it needs to be thin and highly flexible for enabling safe and effective EVT. In order to achieve effective brain cooling, however, the infusate temperature should be as low as possible before entering the circulation [23, 24]. This requirement is highly doubtful to be feasible if standard microcatheters are used [25].

In contrast, the guide catheter remains proximal to the vessel occluding clot. Consequently, pre-reperfusion cold infusion through the guide catheter reaches the ischemic territory solely via collaterals—similar to our experimental simulation (see Fig. 1(a)). Thus, cooling efficacy might critically depend on each patient’s individual clot burden and collateral circulation [26]. The guide catheter, however, is larger and could be sufficiently insulated. Heat-insulated guide catheter–like prototypes for ICCI have already been developed and successfully tested in healthy pigs [27] and in a canine stroke model [28]. Additionally, the larger lumen of the guide catheter would enable uninterrupted ICCI in parallel to EVT.

### Strength and Limitations

The results of our experiment support the feasibility and efficacy of ICCI to rapidly decrease brain temperature [27, 28], much faster than alternative methods of selective brain cooling (e.g., [29]). Hereby, hypothermia duration is limited by the maximally tolerated infusion volume. Considering practicality and ease of translation into clinical application, we confined total ICCI volume to half of each rat’s individual blood volume. Comparable volumes of (intravenous) cold infusion are tolerated by stroke or cardiac arrest patients [22, 30]. In our study, blood pressure and heart rate immediately after ICCI or intravenous cold infusion were lower compared with measurements at 50 min of MCAO (see Table 2). Comparable volume cerebral cold infusion animal studies did not report such changes [5, 28], and stroke patients’ blood pressure increased after high volume intravenous cold infusion [22]. Continuous monitoring of vital parameters instead of far apart punctual measurements may be the key for better understanding of physiologic reactions to ICCI, i.e., differentiating fluid load, systemic, and brain selective hypothermia-mediated effects [22]. Hematocrit decreased by ~ 5% in our study after cold infusion (see Table 2). However, blood gases that are assessed from femoral arterial blood during ICCI do not reflect local conditions in the downstream arterial intracranial circulation. Unfortunately, assessing the latter is technically not feasible in rats. Because local hemodilution during ICCI may negatively affect brain tissue oxygenation, we limited peak flow to 2 mL/min, which equals ~ 50% of rats’ physiological CCA blood flow [18]. In humans undergoing diagnostic angiography, contrast is injected into the ICA or CCA at even higher rates (i.e., 6 and 8 mL/s [31]) which correspond to above 90% of the respective blood flow [32]. However, duration of contrast injection is only 1.5 s compared with 42 s of ICCI at peak flow in our study [31]. Despite all efforts to enhance cooling efficacy, the overall hypothermia duration was less than 20 min in our study. The duration of cerebral cold infusion–induced hypothermia in other small animal studies was shorter, and in some only 3 min [5]. Closed-loop cold autologous blood perfusion might overcome this limitation, but therapeutic heparinization required for preventing emboli formation at the catheter might be deleterious in the acute stroke setting especially when thrombolysis is conducted [33]. Intra-carotid balloon cooling catheter systems have been tested in sheep [29, 34]. Compared with cerebral infusion, their cooling efficacy is low, which renders them unsuitable for fast induction of brain hypothermia [29]. However, due to the absence of fluid load, balloon cooling systems may be used in order to prolong hypothermia beyond ICCI [34]. Unfortunately, balloon catheters for rodent-sized animals do currently not exist. On the other hand, prolonged hypothermia may not even be reasonable: from a practical point of view, in order to liberate limited resources, patients should leave the catheter lab or ICU the soonest possible after EVT. Intra-carotid balloon or any other cooling catheter systems that remain in place would inevitably require extensive supervision plus deep sedation to prevent catheter dislocation, vessel wall dissection or perforation, and other harm to potentially non-compliant and often delirious acute stoke patients. Consequently, before considering to extend hypothermia duration, all efforts should be undertaken to determine other favorable treatment variables. In future studies, strict physiological monitoring, ideally including cerebral hemodynamics and tissue oxygenation, is prerequisite during but not limited to prolonged hypothermia and should be performed alongside with a critical safety assessment focusing on hemorrhagic complications and vessel wall integrity also in old-aged animals and cardiovascular high-risk rat strains.

The aim of our study was to establish the first easily reproducible small animal setup that allows continuous cold infusion from before to after reperfusion and, by reporting all experimental steps in detail, to facilitate its use in future multicenter evaluations. The small sample size of this study does neither allow assessment of neuroprotection nor safety of ICCI. However, we did only observe a single bleeding complication, which we attribute to filament withdrawal rather than cold infusion.

So far, continuous pre- to post-reperfusion intra-arterial cold infusion was successfully realized only in one canine [28] and one primate stroke model [35]. Use of large animal models might be more attractive when studying thermodynamic effects of ICCI due to their larger gyrencephalic brains compared with the small lissencephalic brains of rodents [5]. However, high costs and ethical considerations clearly limit feasibility of large animal cerebrovascular models. Additionally, in pigs, the most widely accepted species for large animal experiments, their rete mirabilis poses a major challenge for simulation of EVT in ischemic stroke [36]. These factors are important considering the need for many more experiments necessary to determine very basic treatment variables such as optimal timing, target temperature, duration, and also composition of cold infusion.

It should be noted that brain temperature curves in our rat experiments closely resemble those of the (non-stroke) pig or (stroke) canine study [27, 28]. Unfortunately, brain temperature has not been measured in the human pilot studies testing cerebral cold infusions during EVT [8–10], temperature trends obtained in the primate stroke model were not reported [35], and lacking information on brain temperature probes’ penetration depth in the canine stroke model does not allow for comparing species related and brain structure specific cooling rates during ischemia and after reperfusion [28]. In our rat experiments, ICCI decreased ipsilateral striatal temperature much faster compared with ipsilateral cortical temperature (see Fig. 5(a, b)). This difference was most pronounced during MCAO (see Fig. 4) when cold infusion reaches the target ischemic tissue via collaterals (see Fig. 6(a)): compared with the distance ICCI must overcome before reaching the cortex via leptomeningeal collateral vessels of the posterior cerebral artery, the distance from the anterior choroidal artery and its branches to deep brain structures adjacent to the striatum is much shorter [37, 38]. Less heat transfer on the way may well explain the more effective cooling of the latter. Most likely, the same also applies for ICCI after reperfusion: distance to the striatum via lenticulostriate branches arising from the proximal MCA is shorter than to the cortex which is supplied by the most distal segments of the MCA [37, 38]. Unfortunately, we did not confirm if and how our approach works in rat strains with poor collaterals such as spontaneous hypertensive rats [37].

Because matching of core body and brain temperature curves during intravenous cold infusion has already been shown in swine and (by our group) even in stroke patients [22, 39], we only conducted one single experiment on systemic cooling with intravenous cold infusion for the purpose of illustration. We did not conceive the present study to evaluate systemic cooling.

## Conclusion

Our experimental setup is the first to successfully implement ICCI-induced brain cooling into the simulated clinical workflow of EVT in a small animal ischemic stroke model with a very early onset of hypothermia induction, i.e., when the guide catheter is placed into the ICA (see Figs. 1(d) and 6(a)). Unlike other experimental and clinical approaches, the suggested “guide catheter approach” promises improved feasibility, timing, and efficacy of regional hypothermia of the ischemic hemisphere. The present method allows local cooling to be initiated before vessel recanalization and continuation of fully temperature-controlled cold infusion until after tissue reperfusion without interruption and unwanted brain temperature fluctuations, which renders our setup the ideal platform to test modifications of the many treatment variables of cerebral cold infusion including timing and target temperature, and to explore their influences on efficacy and safety outcomes before another attempt of a human trial. Our experimental setup may also be used to investigate other types of neuroprotective infusion in the context of EVT.

## Data Availability

Source data will be made available; any other study materials will be made available to other researchers on request to the last author.

## References

[1] Dumitrascu OM, Lamb J, Lyden PD (2016). Still cooling after all these years: meta-analysis of pre-clinical trials of therapeutic hypothermia for acute ischemic stroke. J Cereb Blood Flow Metab : official journal of the International Society of Cerebral Blood Flow and Metabolism.

[2] van der Worp HB, Sena ES, Donnan GA, Howells DW, Macleod MR (2007). Hypothermia in animal models of acute ischaemic stroke: a systematic review and meta-analysis. Brain..

[3] Lyden P, Hemmen T, Grotta J, Rapp K, Ernstrom K, Rzesiewicz T, Parker S, Concha M, Hussain S, Agarwal S, Meyer B, Jurf J, Altafullah I, Raman R, Hess MJ, Mullin A, Jane Hess M, Muranevici G, Piantadosi B, Jimenez-Maggiora G, So JS, Jain S, Diringer M, Derdeyn C, Stern B, Hamilton S, Dietrich D, Becker K, Yenari M, Dirnagl U, Wijman C, Chamorro Á, Janis S, Moy C, Lin F, Song S, Schlick K, Khanolkar P, Edwards NJ, Roldan A, Wilson J, Little A, Lewis P, Neil W, Bruce N, Guzik A, Sohdi A, Herial N, Ovbiagele B, Meyer D, Modir R, Chavez R, Velazquez A, Mayer S, Claassen J, Falo C, Tafreshi G, Neil W, Bruce N, Guzik A, Modir R, Kelly N, Chavez R, Ovbiagele B, Shell E, Dugan G, Kim E, Tanner A, Michel P, Eskandari A, Oddo M, Suys T, Remillard S, Cordier M, Brown R, Jasak S, McCullough L, Brautigam R, Alexandrov A, Sisson A, Albright K, Broessner G, Schmutzhard E, Escioglou E, Jones W, Poisson S, Simpson J, Shah Q, Jonczak K, Bussinger P, Lewandowski C, Berry S, Lundell AM, Miller JB, Cruz-Flores S, Holzer E, Torretta S, Brown D, Heim L, Smith C, Kelley C, Greer D, Marcolini EG, Gilmore EJ, Rutledge N, McBee D, Khanna A, Warren S, Wilsom C, Shushrutha Hedna V, Rosado C, Kizza R, O’Phelan K, Escobar A, Merenda A, Perez Barcena J, Malik A, Collaborators (2016). Results of the ICTuS 2 trial (Intravascular Cooling in the Treatment of Stroke 2). Stroke..

[4] Final Report Summary - EUROHYP-1 (European multicentre, randomised, phase III clinical trial of hypothermia plus best medical treatment versus best medical treatment alone for acute ischaemic stroke). European Commission. 2019. https://cordis.europa.eu/project/rcn/102106/reporting/en. Accessed June 4 2019.

[5] Esposito E, Ebner M, Ziemann U, Poli S (2014). In cold blood: intraarterial cold infusions for selective brain cooling in stroke. J Cereb Blood Flow Metab.

[6] Choi JH, Marshall RS, Neimark MA, Konstas AA, Lin E, Chiang YT, Mast H, Rundek T, Mohr JP, Pile-Spellman J (2010). Selective brain cooling with endovascular intracarotid infusion of cold saline: a pilot feasibility study. AJNR Am J Neuroradiol.

[7] Smith EE, Saver JL, Cox M, Liang L, Matsouaka R, Xian Y, Bhatt DL, Fonarow GC, Schwamm LH (2017). Increase in endovascular therapy in get with the guidelines-stroke after the publication of pivotal trials. Circulation..

[8] Chen J, Liu L, Zhang H, Geng X, Jiao L, Li G, Coutinho JM, Ding Y, Liebeskind DS, Ji X (2016). Endovascular hypothermia in acute ischemic stroke: pilot study of selective intra-arterial cold saline infusion. Stroke..

[9] Peng X, Wan Y, Liu W, Dan B, Lin L, Tang Z (2016). Protective roles of intra-arterial mild hypothermia and arterial thrombolysis in acute cerebral infarction. Springerplus..

[10] Wu C, Zhao W, An H, Wu L, Chen J, Hussain M, Ding Y, Li C, Wei W, Duan J, Wang C, Yang Q, Wu D, Liu L, Ji X (2018). Safety, feasibility, and potential efficacy of intraarterial selective cooling infusion for stroke patients treated with mechanical thrombectomy. J Cereb Blood Flow Metab.

[11] Wu L, Wu D, Yang T, Xu J, Chen J, Wang L, Xu S, Zhao W, Wu C, Ji X (2020). Hypothermic neuroprotection against acute ischemic stroke: the 2019 update. J Cereb Blood Flow Metab.

[12] Wang F, Luo Y, Ling F, Wu H, Chen J, Yan F, He Z, Goel G, Ji X, Ding Y (2010). Comparison of neuroprotective effects in ischemic rats with different hypothermia procedures. Neurol Res.

[13] Alawieh A, Vargas J, Fargen KM, Langley EF, Starke RM, De Leacy R (2019). Impact of procedure time on outcomes of thrombectomy for stroke. J Am Coll Cardiol.

[14] Charbonnier G, Bonnet L, Bouamra B, Vuillier F, Vitale G, Moulin T, Medeiros de Bustos E, Biondi A (2020). Does intravenous thrombolysis influence the time of recanalization and success of mechanical thrombectomy during the acute phase of cerebral infarction?. Cerebrovasc Dis Extra.

[15] Sheth SA, Jahan R, Gralla J, Pereira VM, Nogueira RG, Levy EI, Zaidat OO, Saver JL, for the SWIFT-STAR Trialists (2015). Time to endovascular reperfusion and degree of disability in acute stroke. Ann Neurol.

[16] Meretoja A, Keshtkaran M, Tatlisumak T, Donnan GA, Churilov L (2017). Endovascular therapy for ischemic stroke: save a minute-save a week. Neurology..

[17] Popp A, Jaenisch N, Witte OW, Frahm C (2009). Identification of ischemic regions in a rat model of stroke. PLoS One.

[18] Kenwright DA, Thomson AJ, Hadoke PW, Anderson T, Moran CM, Gray GA, Hoskins P (2015). A protocol for improved measurement of arterial flow rate in preclinical ultrasound. Ultrasound Int Open.

[19] Lee HB, Blaufox MD (1985). Blood volume in the rat. J Nucl Med.

[20] Ding Y, Li J, Rafols JA, Phillis JW, Diaz FG (2002). Prereperfusion saline infusion into ischemic territory reduces inflammatory injury after transient middle cerebral artery occlusion in rats. Stroke..

[21] Ji YB, Wu YM, Ji Z, Song W, Xu SY, Wang Y, Pan SY (2012). Interrupted intracarotid artery cold saline infusion as an alternative method for neuroprotection after ischemic stroke. Neurosurg Focus.

[22] Poli S, Purrucker J, Priglinger M, Ebner M, Sykora M, Diedler J, Bulut C, Popp E, Rupp A, Hametner C (2014). Rapid induction of COOLing in stroke patients (iCOOL1): a randomised pilot study comparing cold infusions with nasopharyngeal cooling. Crit Care.

[23] Konstas AA, Neimark MA, Laine AF, Pile-Spellman J (2007). A theoretical model of selective cooling using intracarotid cold saline infusion in the human brain. J Appl Physiol.

[24] Merrill TL, Mitchell JE, Merrill DR (2016). Heat transfer analysis of catheters used for localized tissue cooling to attenuate reperfusion injury. Med Eng Phys.

[25] Mattingly TK, Lownie SP, Pelz DM (2016). Letter by Mattingly et al. regarding article, “Endovascular hypothermia in acute ischemic stroke: pilot study of selective intra-arterial cold saline infusion”. Stroke..

[26] Tan IY, Demchuk AM, Hopyan J, Zhang L, Gladstone D, Wong K (2009). CT angiography clot burden score and collateral score: correlation with clinical and radiologic outcomes in acute middle cerebral artery infarct. AJNR Am J Neuroradiol.

[27] Choi J, Mangla S, Barone F, Novotney C, Lin E, Pile-Spellman J (2016). Rapid and selective brain cooling and maintenance of selective cooling with intra-carotid cold fluid infusion is feasible and safe. Eur Stroke J.

[28] Caroff J, King RM, Mitchell JE, Marosfoi M, Licwinko JR, Gray-Edwards HL, Puri AS, Merrill TL, Gounis MJ (2020). Focal cooling of brain parenchyma in a transient large vessel occlusion model: proof-of-concept. J Neurointerv Surg.

[29] Cattaneo G, Schumacher M, Maurer C, Wolfertz J, Jost T, Buchert M (2016). Endovascular cooling catheter for selective brain hypothermia: an animal feasibility study of cooling performance. AJNR Am J Neuroradiol.

[30] Kim F, Nichol G, Maynard C, Hallstrom A, Kudenchuk PJ, Rea T, Copass MK, Carlbom D, Deem S, Longstreth WT, Olsufka M, Cobb LA (2014). Effect of prehospital induction of mild hypothermia on survival and neurological status among adults with cardiac arrest: a randomized clinical trial. JAMA : the journal of the American Medical Association.

[31] Raz E, Saba L, Raz E (2015). DSA imaging protocol and technique. Neurovascular imaging - from basics to advanced concepts.

[32] Ackroyd N, Gill R, Griffiths K, Kossoff G, Appleberg M (1986). Quantitative common carotid artery blood flow: prediction of internal carotid artery stenosis. J Vasc Surg.

[33] Solar RJ, Mattingly T, Lownie SP, Meerkin D (2020). Neuroprotection by selective endovascular brain cooling - the TwinFlo catheter. EuroIntervention..

[34] Cattaneo G, Schumacher M, Wolfertz J, Jost T, Meckel S (2015). Combined selective cerebral hypothermia and mechanical artery recanalization in acute ischemic stroke: in vitro study of cooling performance. AJNR Am J Neuroradiol.

[35] Wu D, Chen J, Hussain M, Wu L, Shi J, Wu C et al. Selective intra-arterial brain cooling improves long-term outcomes in a non-human primate model of embolic stroke: efficacy depending on reperfusion status. J Cereb Blood Flow Metab. 2020:271678X20903697. doi:10.1177/0271678X20903697. 10.1177/0271678X20903697 PMC7308521 32126876

[36] Mangla S, Choi JH, Barone FC, Novotney C, Libien J, Lin E, Pile-Spellman J (2015). Endovascular external carotid artery occlusion for brain selective targeting: a cerebrovascular swine model. BMC Res Notes.

[37] Pastor G, Jimenez-Gonzalez M, Plaza-Garcia S, Beraza M, Padro D, Ramos-Cabrer P (2017). A general protocol of ultra-high resolution MR angiography to image the cerebro-vasculature in 6 different rats strains at high field. J Neurosci Methods.

[38] He Z, Yang SH, Naritomi H, Yamawaki T, Liu Q, King MA, Day AL, Simpkins JW (2000). Definition of the anterior choroidal artery territory in rats using intraluminal occluding technique. J Neurol Sci.

[39] Vanden Hoek TL, Kasza KE, Beiser DG, Abella BS, Franklin JE, Oras JJ (2004). Induced hypothermia by central venous infusion: saline ice slurry versus chilled saline. Crit Care Med.

